# Neuropsychiatric Symptoms in Parkinson's Disease After Subthalamic Nucleus Deep Brain Stimulation

**DOI:** 10.3389/fneur.2021.656041

**Published:** 2021-05-04

**Authors:** Weibing Liu, Tatsuya Yamamoto, Yoshitaka Yamanaka, Masato Asahina, Tomoyuki Uchiyama, Shigeki Hirano, Keisuke Shimizu, Yoshinori Higuchi, Satoshi Kuwabara

**Affiliations:** ^1^Department of Neurology, Graduate School of Medicine, Chiba University, Chiba, Japan; ^2^Division of Occupational Therapy, Department of Rehabilitation, Chiba Prefectural University of Health Sciences, Chiba, Japan; ^3^Neurology Clinic, Tsudanuma, Japan; ^4^Department of Neurology, International University of Health and Welfare, Ichikawa, Japan; ^5^Department of Neurological Surgery, Graduate School of Medicine, Chiba University, Chiba, Japan

**Keywords:** Parkinson's disease, deep brain stimulation, quality of life, neuropsychiatric symptoms, UPDRS

## Abstract

**Background:** Indications for subthalamic nucleus deep brain stimulation (STN-DBS) surgery are determined basically by preoperative motor function; however, postoperative quality of life (QOL) is not necessarily associated with improvements in motor symptoms, suggesting that neuropsychiatric symptoms might be related to QOL after surgery in patients with Parkinson's disease.

**Objectives:** We aimed to examine temporal changes in neuropsychiatric symptoms and their associations with QOL after STN-DBS.

**Materials and Methods:** We prospectively enrolled a total of 61 patients with Parkinson's disease (mean age = 65.3 ± 0.9 years, mean disease duration = 11.9 ± 0.4 years). Motor function, cognitive function, and neuropsychiatric symptoms were evaluated before and after DBS surgery. Postoperative evaluation was performed at 3 months, 1 year, and 3 years after surgery.

**Results:** Of the 61 participants, 54 completed postoperative clinical evaluation after 3 months, 47 after 1 year, and 23 after 3 years. Frontal lobe functions, depression, and verbal fluency significantly worsened 3 years after STN-DBS. Non-motor symptoms such as impulsivity and the Unified PD Rating Scale (UPDRS) part I score were associated with QOL after STN-DBS.

**Conclusions:** Frontal lobe functions, depression, and verbal fluency significantly worsened 3 years after STN-DBS. The UPDRS part I score and higher impulsivity might be associated with QOL after STN-DBS.

## Introduction

Parkinson's disease (PD) is a chronic, disabling neurodegenerative disease (1). Recent several studies reported in detail on the importance of non-motor dysfunctions such as cognitive, neuropsychiatric, and autonomic disorders (2–4).

Deep brain stimulation (DBS) is widely used to treat PD patients with motor complications such as wearing off and disabling dyskinesia, for which standard pharmacological treatment is ineffective (2). Ability to perform activities of daily living (ADLs) and quality of life (QOL) are also known to improve after DBS surgery (5–7).

However, our previous study showed that improvements in QOL after DBS surgery are minor compared with improvements in motor symptoms. We have also reported that QOL, as evaluated with the 39-item Parkinson's disease questionnaire (PDQ-39), is not necessarily correlated with motor function after DBS surgery, suggesting that non-motor symptoms might affect QOL after DBS surgery (8).

Some studies have shown that cognitive functions and neuropsychiatric symptoms worsen after DBS surgery in PD patients (9–11). Other studies have demonstrated that postoperative apathy can negate QOL improvement after subthalamic nucleus (STN)-DBS surgery (12). Changes in the depression and anxiety score after DBS surgery are also predictive of QOL after STN-DBS in PD patients (13). The results of these studies suggest that evaluation of the cognitive function and neuropsychiatric symptoms might be helpful in examining QOL after DBS surgery.

This study aimed to assess temporal changes in the cognitive function and neuropsychiatric symptoms (besides motor dysfunctions) after STN-DBS and to examine the relationship between cognitive and neuropsychiatric symptoms and QOL before and after STN-DBS.

## Materials and Methods

### Participants

Between December 2009 and April 2019, we prospectively enrolled 61 PD patients who underwent bilateral STN-DBS at Chiba University Hospital. PD diagnosis was based on the clinical diagnostic criteria of the United Kingdom PD Society Brain Bank (14). All lead (Medtronic, Minneapolis, MN, USA) were implanted bilaterally into the STN in one session under local anesthesia. All participants reported medication-resistant fluctuations and complications in motor function. Before enrollment in the study, participants had been treated with antiparkinson medications and were taking levodopa/carbidopa, dopamine agonists, selegiline, istradefylline, zonisamide, and entacapone. No participants took anticholinergics immediately before or during the study, and motor functions in the “on” and “off” phases while on medications were evaluated with the Unified PD Rating Scale (UPDRS) parts I, II, III, and IV before and after STN-DBS. All postoperative assessments were performed under bilateral ON stimulation. Health-related QOL was assessed with the PDQ-39 Summary Index (SI) before and after STN-DBS, and cognitive functions were evaluated with the Mini Mental State Examination (MMSE), the Frontal Assessment Battery (FAB), and the Japanese version of the Montreal Cognitive Assessment (MoCA-J). The levodopa equivalent dose (LED) of the antiparkinson medications was calculated according to a description elsewhere (15).

In the neuropsychiatric evaluations, we used the verbal fluency test (VFT) to examine verbal fluency by counting the number of words such as animal and the words beginning with a specified word such as “fu,” “a,” and “ni” in Japanese (16). Furthermore, the Barratt Impulsiveness Scale 11 (BIS11) is used to assess the personality/behavioral construct of impulsiveness by a questionnaire composed of 30 items. The “attentional,” “motor,” and “non-planning” impulsiveness can be examined with the BIS11 (17). The Behavioral Inhibition System/Behavioral Activation System (BIS/BAS) scales evaluate impulsivity based on the theory that it can be understood as a joint function of the behavioral approach system (BAS) and the behavioral inhibition system (BIS) by a questionnaire composed of 20 items (18). The Japanese version of the Epworth Sleepiness Scale (JESS) and the rapid eye movement (REM) sleep behavior disorder screening questionnaire (RBD-Q) were used to assess for the presence of sleep disorder. A score of RBD-Q higher than 5 indicates the presence of RBD (19). The Self-Rating Depression Scale (SDS) and an apathy scale were used to evaluate depression and apathy, respectively.

Postoperative evaluations were performed at 3 months, 1 year, and 3 years after STN-DBS.

### Statistical Analysis

All data are expressed as the mean ± standard errors of the mean, and all statistical analyses were performed using SPSS version 23.0 (IBM, Armonk, USA). Analysis of variance (ANOVA) with *post-hoc* analysis (Dunnet's test in this study) were used for comparisons between the baseline (preoperative) scores and the postoperative PDQ-39 Summary Index (SI), UPDRS sub-score, and cognitive functions (MMSE, FAB, and MoCA-J scores) at each follow-up point. ANOVA with *post-hoc* analysis were also used for comparisons between the baseline scores and postoperative neuropsychiatric symptoms (VFT, BIS11, BIS/BAS, JESS, SDS, apathy, and RBD-Q scores) at each follow-up point. Spearman's rank correlation coefficients were calculated to evaluate the relationship between the changes in LED (baseline and postoperative values) and the changes in the score of SDS. Multivariable linear regression analysis was used to determine which cognitive functions, neuropsychiatric symptoms besides the UPDRS sub-score, and LED influenced the QOL (PDQ-39 SI) at baseline and each follow-up point after surgery. Statistical significance was set at *p* < 0.05.

### Ethical Considerations

The Chiba University Hospital Institutional Review Board approved this study. All 61 participants provided written informed consent, obtained during the “on” phase. The ethical standards committee at Chiba University gave approval to implement this study. All participants consented to the use of their examination scores for analysis.

## Results

A total of 61 patients with PD were enrolled in this study (mean age = 65.3 ± 0.9 years, mean disease duration = 11.9 ± 0.4 years). Of the 61 participants, 54 completed the postoperative clinical evaluation after 3 months, 47 after 1 year, and 23 after 3 years. The stimulation parameters were as follows: intensity, 2.7–3.2 V; pulse width, 60 μs; frequency, 130 Hz.

**Figure 1:**
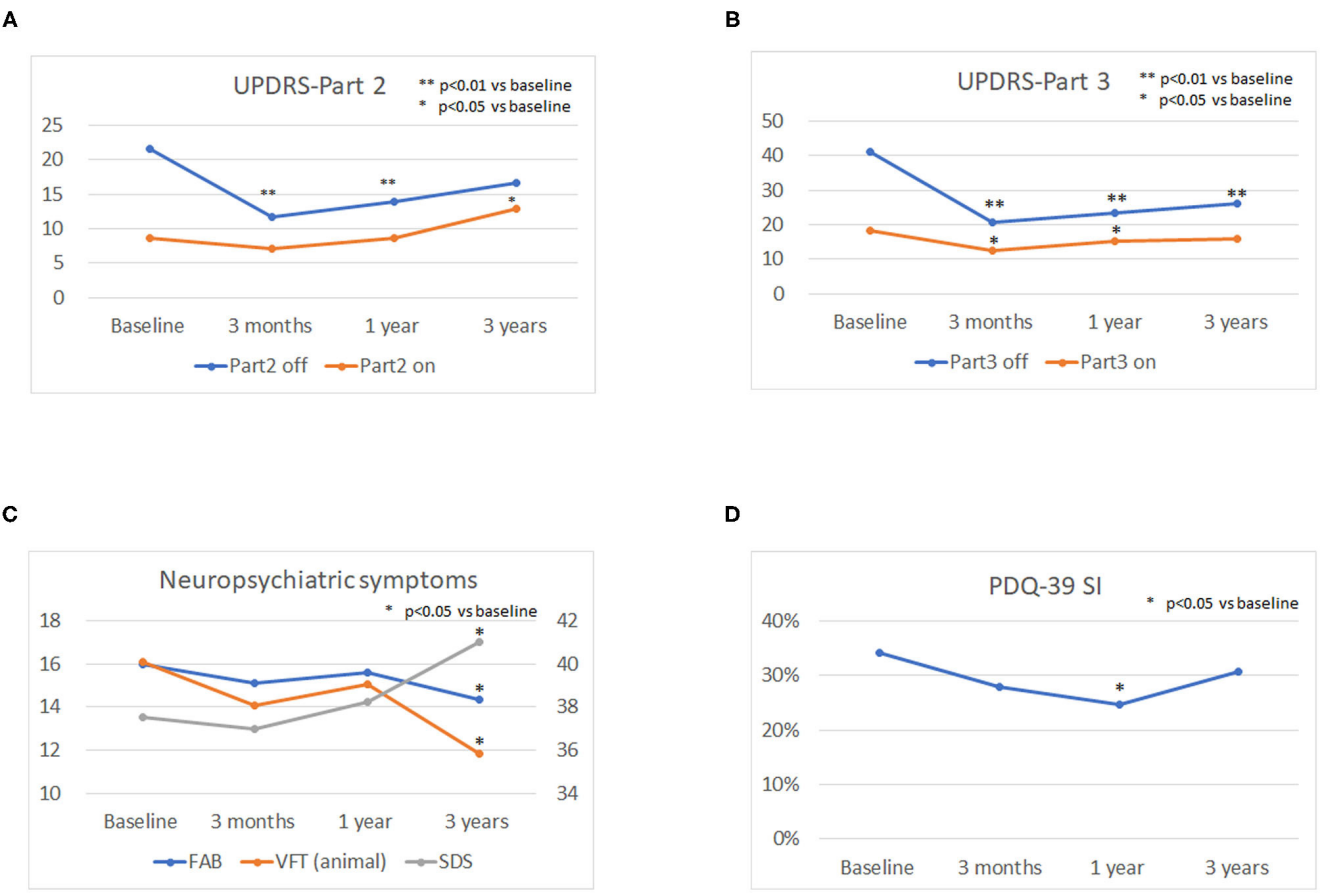
Longitudinal changes in the Unified Parkinson's Disease Rating Scale (UPDRS) parts II and III scores, neuropsychiatric symptoms, and quality of life (QOL). The mean UPDRS parts II and III scores during the off phase **(A,B)** and the UPDRS part IV score decreased significantly (*P* < 0.01) at each follow-up point after surgery compared to the baseline scores. The mean UPDRS part II scores during the on phase did not significantly change 3 months and 1 year after surgery and significantly increased 3 years after surgery **(A)**. The mean UPDRS part III score during the on phase significantly decreased 3 months and 1 year after surgery, and the difference was not significant until 3 years after surgery **(B)**. The depression (SDS) score significantly worsened 3 years after surgery (*P* = 0.021). The verbal fluency test (VFT) score in the animal portion was significantly worse 3 years after surgery than before surgery (*p* < 0.01) **(C)**. The mean PDQ-39 SI significantly decreased from baseline to 1 year after surgery (*p* = 0.015) **(D)**.

The mean LED decreased significantly from baseline dosage at each follow-up point after surgery (*p* < 0.01). The mean UPDRS parts II and III scores during the off phase (Figures 1A,B) and UPDRS part IV decreased significantly (*p* < 0.01) at each follow-up point after surgery compared to the baseline scores. The mean UPDRS part II scores during the on phase did not significantly change 3 months and 1 year after surgery and significantly increased 3 years after surgery (Figure 1A). The mean UPDRS part III scores during the on phase significantly decreased at 3 months and at 1 year after surgery, but the difference was not significant 3 years after surgery (Figure 1B). FAB scores decreased significantly 3 years after surgery (*p* = 0.016; Figure 1C). The cognitive functions as evaluated by the MMSE and MoCA-J did not change significantly from baseline at each follow-up point after surgery. The depression (SDS) score significantly worsened 3 years after surgery (*p* = 0.021). The VFT score in the animal portion was significantly worse 3 years after surgery than before surgery (*p* < 0.01; Figure 1C). The mean PDQ-39 SI significantly decreased from baseline to 1 year after surgery (*p* = 0.015; Figure 1D). An RBD questionnaire score higher than 5 indicates the presence of RBD (19), and because the mean score for the RBD questionnaire was around 4 before and after STN-DBS, the PD patients in this study tended to have RBD-related symptoms. All numerical data on the clinical scales used in this study are represented in Table 1.

**Table 1:** Temporal changes in levodopa equivalent dose, motor functions, cognitive functions, neuropsychiatric functions, and quality of life.

		**Baseline (*n* = 61)**	**3 months (*n* = 54)**	**1 year (*n* = 47)**	**3 years (*n* = 23)**
LED (mg)		1,127.90 ± 39.78	719.12 ± 44.79^**^	670.53 ± 43.11^**^	839.34 ± 72.93^**^
UPDRS	Part I	1.68 ± 0.29	1.22 ± 0.27	1.00 ± 0.19	2.55 ± 0.39
	Part II on	8.95 ± 0.95	7.22 ± 0.94	8.58 ± 0.99	13.05 ± 1.675^*^
	Part II off	22.83 ± 1.13	11.63 ± 1.73^**^	13.48 ± 2.06^**^	17.23 ± 1.87
	Part III on	19.71 ± 1.22	12.62 ± 1.205^*^	14.81 ± 1.405^*^	16.23 ± 1.79
	Part III off	42.66 ± 1.86	20.71 ± 2.26^**^	22.68 ± 2.80^**^	26.95 ± 2.95^**^
	Part IV	8.15 ± 0.38	3.45 ± 0.28^**^	3.14 ± 0.26^**^	4.10 ± 0.27^**^
QOL	PDQ-39 SI (%)	34.00 ± 2.64	28.71 ± 2.29 (−18.15% decrease from baseline)	23.45 ± 2.455^*^ (−27.54% decrease from baseline)	31.68 ± 3.44 (−10.07% decrease from baseline)
Cognitive and neuropsychiatric functions	MMSE	28.63 ± 0.23	28.33 ± 0.34	28.78 ± 0.25	27.95 ± 0.61
	FAB	16.40 ± 0.24	15.09 ± 0.49	15.50 ± 0.39	14.37 ± 0.675^*^
	MoCA-J	26.05 ± 0.44	26.0 ± 0.61	25.65 ± 0.53	24.65 ± 0.85
	VFT (Katakana)	24.11 ± 1.97	24.47 ± 1.93	21.94 ± 2.77	21.53 ± 2.01
	VFT (animal)	16.36 ± 1.15	13.70 ± 1.11	15.08 ± 1.04	12.11 ± 1.63^**^
	BIS11	53.50 ± 3.34	48.53 ± 2.07	53.07 ± 2.64	56.44 ± 1.85
	BIS/BAS	47.78 ± 2.07	46.27 ± 1.95	49.71 ± 1.96	49.29 ± 2.85
	JESS	7.21 ± 1.24	7.47 ± 1.54	9.88 ± 1.60	8.18 ± 1.36
	SDS	34.74 ± 1.13	35.40 ± 1.43	37.69 ± 1.63	41.24 ± 1.45^*^
	Apathy	4.42 ± 0.93	7.80 ± 1.46	5.88 ± 1.16	6.29 ± 1.14
	RBDQ	4.11 ± 0.69	3.87 ± 0.49	4.19 ± 0.52	3.94 ± 0.53

*Apathy, apathy scale; BIS11, Barratt impulsiveness scale 11; BIS/BAS, behavioral inhibition system/behavioral activation system; FAB, Frontal Assessment Battery; JESS, Japanese version of the Epworth Sleepiness Scale; LED, levodopa equivalent dose; MMSE, Mini Mental State Examination; MoCA-J, Japanese version of Montreal Cognitive Assessment; PDQ-39, 39-item Parkinson's disease questionnaire; QOL, quality of life; RBDSQ, REM sleep behavior disorder screening questionnaire; SDS, Self-Rating Depression Scale; SI, Summary Index; UPDRS, Unified Parkinson's Disease Rating Scale; VFT, verbal fluency test*.

** *p < 0.01 vs. preoperative values*;

* *p < 0.05 vs. preoperative values*.

### Correlation Between the Changes in LED and the Changes in the Score of SDS

The correlation coefficients between the changes in LED and the changes in SDS at each follow-up point were 0.136 (*p* = 0.596; baseline, 3 months after DBS), −0.051 (*p* = 0.828; baseline, 1 year after DBS), and 0.066 (*p* = 0.847; baseline, 3 years after DBS).

### Multivariable Linear Regression Analysis

At baseline, a higher UPDRS part III score during the off phase (standardized β = 0.373, *p* = 0.046), a higher part IV score (standardized β = 0.335, *p* = 0.018), and a higher BIS11 score (standardized β = 0.576, *p* = 0.022) were significantly associated with higher PDQ-39 SI. At 3 months after DBS, a higher UPDRS part I score (standardized β = 0.594, *p* = 0.022) and a higher BIS/BAS score (standardized β = 0.822, *p* = 0.005) were significantly associated with higher PDQ-39 SI. At 1 year after DBS, although no parameters were associated with PDQ-39 SI, a higher BIS11 score (standardized β = 0.590, *p* = 0.064) tended to be associated with higher PDQ-39 SI. At 3 years after DBS, a higher UPDRS part I score (standardized β = 0.686, *p* = 0.014) was associated with higher PDQ-39 SI.

The cognitive functions as evaluated by the MMSE, FAB, and MoCA did not significantly contribute to pre- and postoperative QOL (Table 2).

**Table 2:** Standardized beta values of the factors determining quality of life at each follow-up point.

**Quality of life at each follow-up point**	**Factors determining quality of life at each follow-up point**	**Standardized β**	***p***
PDQ-39 SI before DBS surgery	BIS11 (impulsivity)	0.576	0.022
	UPDRS part III during the off phase	0.373	0.046
	UPDRS part IV	0.335	0.018
PDQ-39 SI 3 months after DBS surgery	UPDRS part I	0.594	0.022
	BIS/BAS	0.822	0.005
PDQ-39 SI 1 year after DBS surgery	None		
PDQ-39 SI 3 years after DBS surgery	UPDRS part I	0.686	0.014

*PDQ-39 SI, 39-item Parkinson's disease questionnaire summary index; DBS, deep brain stimulation; BIS11, Barratt Impulsiveness Scale 11; UPDRS, Unified PD Rating Scale; BIS/BAS, behavioral inhibition system/behavioral activation system*.

## Discussion

Although motor complications dramatically improved after DBS surgery, many PD patients are not necessarily satisfied with their QOL (8), suggesting that QOL after the surgery may be affected instead by non-motor parameters such as cognitive function and neuropsychiatric symptoms. Thus, we aimed to assess the temporal changes in cognitive function and neuropsychiatric symptoms and to determine which cognitive functions and neuropsychiatric symptoms were related to QOL after DBS surgery.

The present results revealed that the scores of UPDRS parts II and III during the off phase and the UPDRS part IV score decreased significantly from baseline after STN-DBS, which are compatible with the well-known clinical effect of STN-DBS on motor dysfunctions (1). The present study also revealed that frontal lobe function, depression, and verbal fluency significantly worsened 3 years after STN-DBS. Furthermore, preoperative QOL was significantly associated with the severity of preoperative motor symptoms (UPDRS part III during the off phase), motor complication (UPDRS part IV), and impulsivity (BIS 11), suggesting that worse motor complications and impulsivity led to worse QOL preoperatively. Postoperative QOL 3 months after surgery was significantly associated with non-motor symptoms (UPDRS part I) and impulsivity as evaluated by BIS/BAS, suggesting that worse non-motor symptoms and impulsivity led to worse QOL 3 months after surgery. Although no clinical parameters were significantly associated with postoperative QOL 1 year after surgery, postoperative QOL at 3 years was significantly associated with non-motor symptoms (UPDRS part I) and impulsivity as evaluated by BIS11, suggesting that worse non-motor symptoms and impulsivity led to worse QOL postoperatively 3 months after surgery.

In terms of a decline in frontal lobe function 3 years after STN-DBS, our previous report also revealed that the FAB score tended to decrease 3 years after STN-DBS, without statistical significance (8). Because this study included a larger number of PD patients compared to our previous study (8), the decline in frontal lobe function 3 years after STN-DBS compared to baseline probably became significant in this study. Although it is difficult to identify whether the decline in FAB score 3 years after STN-DBS was attributable to the effect of surgery or natural disease progression, our previous report revealed that PD patients who underwent STN-DBS showed a decrease of cerebral blood flow in the prefrontal and cingulate cortex 4.3 ± 1.1 months (range = 2.9–6.6 months) after STN-DBS (10). Hence, frontal lobe functions should be carefully examined for more than 3 years after STN-DBS.

This study also showed that depression worsened 3 years after STN-DBS. Although a reduction in LED might partially contribute to the worsening of depression, dopamine withdrawal syndrome usually occurs less than 1 year after STN-DBS (20). Furthermore, the relationships between the changes in LED and the changes in the depression scale did not show significant correlations in this study. We do not know the exact reason why the depression score worsened significantly only at 3 years after STN-DBS, nor whether the worsening of depression scores is attributed to the effect of STN-DBS or is a natural disease progression.

Verbal fluency is well-known to worsen after STN-DBS (21). Although we do not know why the worsening of verbal fluency was significant only at 3 years after STN-DBS, chronic changes in the microlesion effect of the electrode trajectory might contribute to verbal fluency (22).

In terms of the associations between QOL and motor, cognitive, and neuropsychiatric symptoms, deteriorated motor symptoms during the off phase and severe motor complications were significantly associated with worse QOL preoperatively, which was reasonable because the indication for STN-DBS is the presence of motor complications accompanied by a severely deteriorated ADL during the off phase. The higher impulsivity was also associated with worse QOL preoperatively. Although we do not know the exact reason why a higher impulsivity leads to worse QOL preoperatively, a possible explanation might be that patients with higher impulsivity were more strongly dissatisfied with preoperative mobility and ADL (23).

On the contrary, postoperative QOL was associated with non-motor symptoms, as evaluated by UPDRS part I, rather than motor symptoms or ADL. The significant positive associations between the UPDRS part I score and the PDQ-39 SI were found 3 months and 3 years after STN-DBS. Because UPDRS part I includes questions on cognitive functions, hallucination, depression, and motivation, we might say that non-motor symptoms partially contribute to QOL after STN-DBS in PD patients. Although there are few studies examining the associations between the UPDRS part I score and PDQ-39 SI before and after STN-DBS, numerous studies have reported on the effect of STN-DBS on cognitive and neuropsychiatric symptoms in PD patients (24–27). However, the effect of STN-DBS on cognitive functions and neuropsychiatric symptoms may differ, and a detailed interpretation of the positive associations between the UPDRS part I score and PDQ-39 SI is difficult. It is interesting that impulsivity, as evaluated by BIS/BAS, was significantly associated with postoperative QOL 3 months after surgery, whereas BIS11 was significantly associated with postoperative QOL 3 years after surgery. BIS11 classified impulsivity into “attentional,” “motor,” and “non-planning” impulsiveness (16), whereas BIS/BAS evaluate impulsivity based on the theory that it can be understood as a joint function of the behavioral approach system (BAS) and the behavioral inhibition system (BIS) (17). Despite the marked reductions in LED after surgery, impulsivity was significantly associated with postoperative QOL. Mosley et al. reported that greater connectivity of the stimulation site with the frontostriatal network was related to greater postoperative impulsiveness and disinhibition (28). Although we do not know the exact reason why BIS/BAS was associated with postoperative QOL 3 months after surgery, whereas BIS11 was associated with postoperative QOL 3 years after surgery, a higher impulsivity might contribute to postoperative QOL, and impulsivity should be carefully examined after surgery.

Several limitations of this study should be considered. One major limitation is that not all patients completed the follow-up evaluations. Some patients at each follow-up point are now under investigation. Therefore, the smaller number of patients at each follow-up point compared to baseline does not indicate a high prevalence of the dropout rate in this study. Another limitation was that, although the postoperative score of UPDRS part I was associated with QOL, the cognitive functions (MMSE, FAB, and MoCA-J) and neuropsychiatric symptoms, except for impulsivity, were not associated with postoperative QOL at each follow-up point. One possible explanation for this discrepancy might be that the MMSE, FAB, SDS, and the apathy scale are not specific scales for PD patients, whereas UPDRS part I is specific for PD patients. However, these points should be further examined with a larger number of PD patients. It should also be addressed that the present study lacks a control group, which means that the postoperative changes in the cognitive and neuropsychiatric symptoms and their association with QOL may not solely result from STN-DBS, but the degeneration itself also has a contribution. However, it might be practically very difficult to compare advance-stage PD patients undergoing DBS surgery with PD patients who receive only the best medical treatment. Advance-stage PD patients who do not undergo DBS surgery usually have cognitive impairments and severe neuropsychiatric symptoms or less severe motor complications in which DBS surgery is not indicated. Because DBS is a clinically established surgery for PD patients suffering from motor complications, randomization of PD patients into a DBS group and a best medication group is currently difficult. Furthermore, the selection of a stimulation target might also result in selection bias. In our hospital, STN-DBS are basically administered to PD patients who were suffering from a wearing-off phenomenon rather than dyskinesia and *vice versa* for globus pallidus pars interna (GPi)-DBS. Since non-motor symptoms might be more prevalent and severe in PD patients receiving GPi-DBS compared to those receiving STN-DBS, selection of the stimulation target might lead to selection bias.

Nevertheless, the present results might be important because temporal changes in the cognitive and neuropsychiatric symptoms and their association with QOL were provided in this study. Since many of the previous studies usually examined non-motor symptoms at only one point, our results might be helpful for clinical practice.

## Conclusion

Frontal lobe functions, depression, and verbal fluency significantly worsened 3 years after STN-DBS. The UPDRS part I score and a higher impulsivity might be associated with QOL after STN-DBS.

## Data Availability Statement

The original contributions presented in the study are included in the article/supplementary material, further inquiries can be directed to the corresponding author/s.

## Ethics Statement

The studies involving human participants were reviewed and approved by Chiba University Hospital Institutional Review Board. The patients/participants provided their written informed consent to participate in this study.

## Author Contributions

All authors listed have made a substantial, direct and intellectual contribution to the work, and approved it for publication.

## Conflict of Interest

The authors declare that the research was conducted in the absence of any commercial or financial relationships that could be construed as a potential conflict of interest.
